# Local striatal reward signals can be predicted from corticostriatal connectivity

**DOI:** 10.1016/j.neuroimage.2017.07.042

**Published:** 2017-10-01

**Authors:** Peter Smittenaar, Zeb Kurth-Nelson, Siawoosh Mohammadi, Nikolaus Weiskopf, Raymond J. Dolan

**Affiliations:** a Wellcome Trust Centre for Neuroimaging, Institute of Neurology, University College London, London, WC1N 3BG, UK; b Max Planck-University College London Centre for Computational Psychiatry and Ageing Research, London, WC1B 5EH, UK; c Department of Systems Neuroscience, Medical Center Hamburg-Eppendorf, Hamburg, Germany; d Department of Neurophysics, Max Planck Institute for Human Cognitive and Brain Sciences, 04103, Leipzig, Germany

## Abstract

A defining feature of the basal ganglia is their anatomical organization into multiple cortico-striatal loops. A central tenet of this architecture is the idea that local striatal function is determined by its precise connectivity with cortex, creating a functional topography that is mirrored within cortex and striatum. Here we formally test this idea using both human anatomical and functional imaging, specifically asking whether within striatal subregions one can predict between-voxel differences in functional signals based on between-voxel differences in corticostriatal connectivity. We show that corticostriatal connectivity profiles predict local variation in reward signals in bilateral caudate nucleus and putamen, expected value signals in bilateral caudate nucleus, and response effector activity in bilateral putamen. These data reveal that, even within individual striatal regions, local variability in corticostriatal anatomical connectivity predicts functional differentiation.

## Highlights

• We use diffusion imaging to predict striatal reinforcement learning BOLD responses.
• Structural cortico-striatal connectivity can explain intra-regional BOLD variability.
• These results suggest a fine functional parcellation based on afferent connectivity.

## 1 Introduction

The basal ganglia, the central structures in reward-guided action selection, exhibit a remarkably intricate architecture whereby inputs from cortex are topographically organized into multiple cortico-striatal loops (Alexander et al., 1986). Rather than a division into neatly segregated pathways, axons from multiple cortical regions converge in overlapping parts of the striatum (Averbeck et al., 2014, Haber, 2010). This places the striatum at a crossroads of information processing thought to drive, amongst other functions, reward-guided behaviors (Averbeck et al., 2014, Haber and Behrens, 2014). This arrangement is somewhat at odds with an otherwise rigid anatomical parcellation of the striatum into nucleus accumbens, caudate nucleus and putamen (Voorn et al., 2004). Here we ask whether knowing the corticostriatal inputs to each voxel of the striatum allows us to predict functional activity within that voxel. If we can do so even *within* classical subregions of the striatum – the caudate nucleus and the putamen – then this suggests that a detailed knowledge of structural connectivity can provide a more detailed guide to local function than does anatomy alone.

We tested our hypothesis using a methodology first reported in a study within the visual domain (Saygin et al., 2012). This previous study reported an accurate prediction of functional responses to faces versus scenes for individual voxels in the fusiform gyrus based on structural connectivity fingerprints of these very same voxels. Although this approach has been extended to visual responses in other regions of cortex (Osher et al., 2015), to the best of our knowledge it has not been applied to higher cognitive functions or to an examination of subcortical structures. Given the great diversity of inputs into the striatum—spanning most of cortex (Alexander et al., 1986)—its subregions are particularly well-suited for an examination of such structure-function relationships. Specifically, we examined the caudate nucleus and putamen during an instrumental reinforcement learning task using functional and diffusion-weighted magnetic resonance imaging (MRI). To validate our approach we examined motor effector activity related to hand and foot actions. We then used this method to predict individual intra-region variability in the expression of reward and expected value signals from individual corticostriatal connectivity profiles, finding a dependence of function on each voxel's distinct pattern of cortical connectivity.

## 2 Materials and methods

### 2.1 Participants

Twenty-four adults participated in the experiment (14 female, 10 male; age range 18–36 years; mean ± SD = 22.5 ± 4.5 years). All participants were right hand dominant, had no history of psychiatric or neurological disorder, were not taking any medication known to affect neural or cognitive function, had normal or corrected-to-normal vision and passed the safety requirements to enter a MRI scanner. All subjects provided written informed consent prior to the start of the experiment, which was approved by the Research Ethics Committee at University College London (UK). One further subject was excluded due to excessive movement (images could not be realigned successfully).

### 2.2 Overview of the approach

We tested the hypothesis that corticostriatal input into the caudate nucleus and putamen reliably predicts functional responses during instrumental learning. To do so we estimated, for each voxel in bilateral caudate nucleus and putamen, functional activation to motor responses, reward and expected value activations during a 2-armed bandit task. These same voxels were also characterized in terms of their structural connectivity to 148 cortical regions using diffusion imaging and probabilistic tractography. We could then predict functional activation from corticostriatal structural connectivity using a leave-one-out cross-validation (LOOCV) procedure (Saygin et al., 2012). All these analyses were performed in subject space, with only summary statistics for each participant taken to the group level. All reported p-values are two-tailed.

### 2.3 Task

**Fig. 1:**
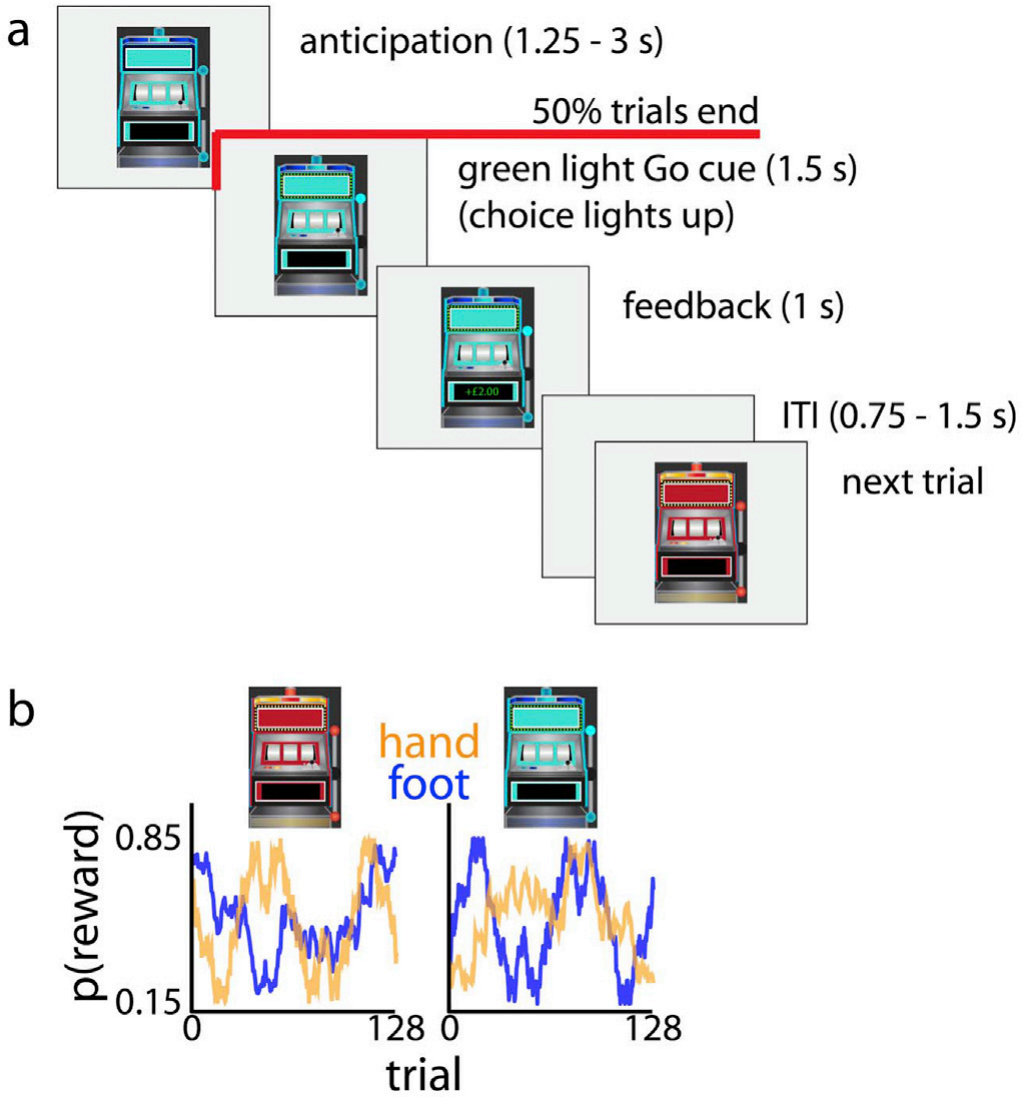
Reinforcement learning task involving right hand and right foot responses. (a) Task design. On half the trials (‘abort’ trials) the slot machine disappeared before the Go signal and the next trial started; on the other half (‘response’ trials) lights on the slot machine would turn green, serving as a Go signal; participants responded by pressing force-sensitive buttons with either their right hand or foot. Feedback was then presented consisting of either “+ £2.00” in green, or “+ £0.00” in red. (b) The probability of obtaining the reward varied over time per response, and per slot machine. This meant participants were required to track 4 random walks that varied between p (reward) of 0.15 and 0.85.

The task required participants track stimulus-specific action values and this enabled us to probe how these action values are represented and updated in neural structures during feedback. Participants had to learn two separate two-armed bandits which were distinguished by their color (red or blue; see Fig. 1). On each trial, one of these two slot machines was presented to the participant, and on half the trials a response was required using either right index finger or right ball of the foot on a force-sensitive sensor. Binomial feedback was then presented which indicated a reward or no-reward. The probability of reward given a bandit s and action a, $p\left(r|s_{i},a_{j}\right)$ where i∈{1,2} and j∈{1,2}, changed slowly over trials, forcing participants to continue to explore throughout the experiment so as to maximise the total reward obtained.

Participants came to the laboratory for a practice session before the scanning session. The interval between practice and scanning session ranged between 1 and 20 days (mean ± SD = 7 ± 4.4 days). At the practice session, participants performed a full set of 512 trials to accustom themselves with the task and force buttons. A different set of reward probabilities was used each day but otherwise the parameters of the experiment were identical. In the experiment proper, participants performed 512 trials (approximately 42 min) consisting of 128 red-abort, red-response, blue-abort, and blue-response trials each (Fig. 1). The order of these four trial types was randomly determined and only constrained such that no trial type occurred for more than 3 trials in a row.

#### 2.3.1 Reward probabilities

The $p_{t}\left(r|s_{i},a_{j}\right)$, where t indicates trial number, was generated by a Gaussian random walk for each action a and stimulus s as follows:

$$p_{t+1}\left(r|s_{i},a_{j}\right)=p_{t}\left(r|s_{i},a_{j}\right)+N\left(0,0.01\right)$$

where for the first trial the probability was randomly drawn from *U(0.15,0.85)*. The walks were not generated anew for each participant—rather, one set of two pairs was used for each participant's practice, and one set was used for each participant's scanning session. However, the assignment of these two pairs to the red and blue slot machine was randomized, and the subsequent assignment of random walk to the two available actions was also randomized. This meant that volatility and availability of reward were matched between participants. The walks were constrained in their upper (0.85) and lower (0.15) values and in their mean value (between 0.4 and 0.6). The highest correlation between any two of the four walks was 0.38, forcing participants to learn about the value of each option through trial-and-error rather than inferring the value of one option based on changes in the other.

#### 2.3.2 Trial design

Examining value representations in the BOLD signal at both choice and outcome phase is challenging due to the sluggishness of the BOLD response. We considered two trial designs to alleviate this issue: a slow design where choice and feedback events are separated by at least 8 s (Behrens et al., 2008), and a fast design in which half the trials are cancelled at any point between choice and feedback phase (Guitart-Masip et al., 2012). Pilot data with both designs (data not shown) suggested participants were more accurate at learning reward probabilities in the fast design, possibly due to task disengagement when participants are faced with long pauses. Also, a slow design might lead to non-striatal learning mechanisms dominating behavior, whereas we were specifically interested in such striatal mechanisms (Foerde et al., 2012). We thus opted for the fast design. In this paper we do not report correlates of action values during choice as we were unable to reliably observe its neural correlates in the striatal regions; we only examine motor responses, expected value at outcome and reward responses.

### 2.4 Reinforcement learning models

We used temporal difference (TD) reinforcement learning models to model participants’ behavior and estimate quantities that might be represented in the BOLD signal in the striatum, most notably rewards and action values. Each slot machine *i* defines a state *s*_*i*_ where two actions *a*_*j*_ are available. The reward *r* on trial *t* can be either 0 or 1. The value of action *j* in state *i* is updated after feedback by:

$$Q_{s_{i},a_{j}}\left(t+1\right)=Q_{s_{i},a_{j}}\left(t\right)+\alpha *\partial \left(t\right)$$

where α = 0 for all states and actions that did not occur on trial t-1. As the reward probabilities change independently for each state and action, the participant only learns about the chosen action in the current state, rather than inferring changes in value for non-chosen state-action pairs in a ‘model-based’ way (except for value decay—see below). ∂(t) represents the RPE at trial t, defined as

$$\partial \left(t\right)=r\left(t\right)-Q_{s_{i},a_{j}}\left(t\right)$$

The probability of each action given these cached values ∂(t) are then given by the softmax equation with inverse temperature β:

$$p\left(a_{j},s_{i}\right)=e^{\beta *Q_{s_{i},a_{j}}}/\sum_{k=1}^{2}e^{\beta *Q_{s_{i},a_{k}}}$$

We used an expectation maximization (EM) approach to simultaneously fit parameters at the level of participants and population (Guitart-Masip et al., 2012).

In addition to this basic model with a learning rate and inverse temperature we examined a number of more complex models that might provide a better explanation for the data. For each of these models we estimated the negative log-likelihood and Bayesian Information Criterion (BIC) to select the model that optimally described the participant's behavior on this task. The additional parameters are described in Table 1. All parameter combinations were tested.

**Table 1:** Additional parameters for the reinforcement learning model.

Parameter name	Description
Negative learning rate	Separate learning rate for negative and positive feedback
Effector bias	A fixed bias towards hand or foot responses
Lapse rate	A value that constrains the softmax between ε and 1-ε rather than 0 and 1 to account for occasional lapses
Decay	Implements the notion that unsampled actions do not maintain their value but decay back to 0.5. The parameter describes the time constant of exponential decay.
Perseverance	A tendency to stick with the same action for a given stimulus, irrespective of value.

### 2.5 Magnetic resonance imaging

All imaging was performed at 3 T. For each participant we acquired 1.5 mm isotropic restricted volume T2*-weighted echo-planar imaging (EPI) data during task performance, 0.8 mm isotropic whole-brain multi-parameter maps (MPMs) consisting of a T1-, proton density- and magnetization transfer-weighted (MT) volume, 1.5 mm isotropic whole-brain diffusion weighted images, and B0 field maps to correct for field inhomogeneity for the EPI data. The MPMs were acquired to allow for manual segmentation of subcortical structures, though the work presented here makes no use of these manually segmented regions (more details below). The parameters of these scans are detailed in Table 2. We also acquired a single whole-brain volume using otherwise identical settings for the EPI sequence. Cardiac rate was recorded using an MRI-compatible pulse oximeter (Model 8600 F0, Nonin Medical), and respiration was monitored using a pneumatic belt positioned around the abdomen. We processed these cardiorespiratory data as described in the literature (Hutton et al., 2011) and included them as regressors of no interest in the first-level general linear models (see below).

**Table 2:** MRI acquisition parameters.

Sequence	Parameters
B0 field map	Double echo FLASH sequence (matrix size = 64 × 64; 64 slices; spatial resolution = 3 × 3 × 3 mm^3^; gap = 1 mm; short TE = 10 ms; long TE = 12.46 ms; TR = 1020 ms) to correct EPI images for distortion in the B0 field (Weiskopf et al., 2006).
Functional, EPI	Restricted volume, 44 slices (40 in slab with 10% oversampling), FoV read 192 mm, transverse slices tilted 20°, anterior-posterior phase encoding, 12% phase oversampling, 10% slice oversampling, 40 slices per slab, voxel size 1.5 mm isotropic, TR = 78 ms (volume TR = 3432 ms, i.e. 44 slices * 78 ms), TE = 37.3, GRAPPA2 along phase encoding (full set of external reference scans with 144 PE ref. lines, 44 3D ref. lines), 180–185 vol per block depending on duration of block over 4–10 min blocks in total (Lutti et al., 2012).
Multi-parameter maps	Proton density (PD)-weighted, T1-weighted, and magnetization transfer (MT)-weighted images at 0.8 mm isotropic resolution for each participant using multi-echo 3D FLASH, TR = 25 ms, TE = [2.34, 4.64, 6.94, 9.24, 11.54, 13.84, 16.14, 18.44] ms, FOV read 256 mm, FOV phase 87.5%, slice partial Fourier 6/8, GRAPPA acceleration 2 (Dick et al., 2012, Helms et al., 2008). Flip angle for PD and MT was 6°, for T1 21°. A B1-map was acquired using a 3D SE/STE EPI method to correct for the effects of inhomogeneous radio-frequency excitation on the quantitative maps (Lutti et al., 2012). Total time of acquisition was ∼40 min.
Diffusion-weighted, whole-brain	Whole-brain 1.5 × 1.5 × 1.5 mm^3^ resolution diffusion-weighted images with settings similar to the Human Connectome Project (Sotiropoulos et al., 2013, Van Essen et al., 2012). Three shells (b = 900/1800/2700) for both right-left and left-right phase-encoding directions. Each of these 6 scans contained 10 images with no diffusion weighting (b = 0) and 100 directions spread out over a full sphere. We used multiband 3 but no further acceleration. Acquisition time was 10 min 20 s for each of the 6 scans. No phase oversampling, 75 transverse slices, FoV read 192 mm, FoV phase 100%, slice thickness 1.5 mm with 0 distance between slices, TR 5440, TE 130 ms. We additionally acquired two b0 images with identical settings, but phase encoding along anterior-posterior and along posterior-anterior respectively. These additional phase encoding directions were included in estimating distortions along the phase encoding direction.

#### 2.5.1 Multi-parameter maps processing

Fully quantitative maps of the MR parameters MT, R1, PD and R2* were extracted from the acquired data as described previously (Helms et al., 2008, Weiskopf et al., 2013). We extracted a brain mask in structural space from the T1w image using BET implemented in FSL (Smith, 2002).

#### 2.5.2 Semi-automatic segmentation of basal ganglia substructures

Whereas the striatum can be reasonably defined using automated algorithms, other parts of the basal ganglia require manual segmentation. These regions comprised the globus pallidus pars interna (GPi) and externa (GPe), subthalamic nucleus (STN) and substantia nigra and ventral tegmental area (SN/VTA). We used FSL FIRST to automatically segment the bilateral caudate and putamen (Patenaude et al., 2011), and ITK-SNAP to correct the automatic segmentation as well as segment the remaining regions (Yushkevich et al., 2006). Note that segmentation was performed bilaterally for each participant as it is unclear to what extent basal ganglia function is lateralized (Scholz et al., 2000). The current paper only used the putamen and caudate nucleus maps, excluding the nucleus accumbens as probabilistic tractography from this region of interest was found to be challenging. The main reason is that the nucleus accumbens is relatively small and does not have the same spatial extent as the other two striatal regions. This provides fewer opportunities for true variance in structural connectivity patterns. All segmented regions are available as probability maps in MNI space at 0.8 mm isotropic resolution (http://neurovault.org/collections/1380/).

#### 2.5.3 Automatic segmentation of cortex using FreeSurfer

To obtain cortical targets for tractography we used FreeSurfer's RECON-ALL pipeline to generate 148 cortical labels in structural (participant) space following the Destrieux atlas (Destrieux et al., 2010, Fischl, 2012). These were transformed into volumetric ROIs. Two participants lacked 1 and 3 labels, respectively, so these were added as empty ROIs for tractography (see below). The FreeSurfer segmentation pipeline has been described in detail elsewhere (Fischl et al., 2004).

#### 2.5.4 FMRI preprocessing

We analyzed the fMRI data in SPM8 (Wellcome Trust Centre for Neuroimaging, UCL, London; www.fil.ion.ucl.ac.uk/spm). The images were corrected for signal bias at low spatial frequencies (due to the 32 channel radio-frequency receive coil), realigned to the first functional image and distortion corrected using the B0 field maps. We did not apply slice time correction as we used a 3D EPI sequence. The first functional image was coregistered to the MT image for its superior subcortical performance in white- and grey-matter segmentation compared to T1-weighted images (Helms et al., 2009) and these transformation parameters were then applied to all restricted-volume functional images to bring them into structural space. Notably, SPM's coregistration of the restricted-volume EPI to the MT image worked well, obviating the need for an intermediate step involving the whole-brain EPI images. For additional analyses of group-level responses we applied normalization parameters to the functional images to bring them into MNI space and applied a 6 mm full-width-half-maximum (FWHM) smoothing kernel. All participant-level statistics were performed on voxels within an explicit mask (rather than the more commonly used implicit mask) to prevent brain voxels with low signal from being excluded. The explicit mask for structural (i.e. subject) space was constructed by restricting the whole-brain mask (see multi-parameter maps) to the volume of the EPI sequence using SPM's IMCALC.

#### 2.5.5 FMRI general linear model

The preprocessed images were analyzed in an event-related design using a general linear model (GLM). The first model contained 8 explanatory variables of interest (EVs) defined at the onset of the visual stimulus (2 identical EVs), the ‘go’ cue when choosing hand (1 EV) or foot (1 EV), the onset of feedback after choosing hand (2 identical EVs), and the onset of feedback after choosing foot (2 identical EVs). A number of identical EVs were entered to be able to add multiple, non-orthogonalized parametric modulators to specific events. These parametric modulators were the Q-value for the hand and foot at visual stimulus; the Q-value for the hand and foot on the respective response EVs, and whether reward was received for the respective feedback EVs.

We added the following nuisance regressors: 1 regressor for trials where no response was recorded in the 1500 ms response window, 1 regressor when the trial was aborted, 6 movement regressors produced by the realignment procedure, 14 physiological regressors for cardiac and respiratory variables (Hutton et al., 2011), and 3 block regressors covering run 1 to 3, respectively. The 4th block was subsumed in the constant of the design matrix. The GLM was estimated separately for each participant. All EVs (but not physiological regressors) were convolved with a canonical hemodynamic response function (Friston et al., 1995).

#### 2.5.6 Diffusion weighted imaging preprocessing

The diffusion data was preprocessed using FSL (Smith et al., 2004). We estimated the distortions along phase-encoding directions by entering 8 b0 images into TOPUP (Andersson et al., 2003). The field coefficients were then supplied to EDDY, which corrects for the phase-encoding distortion, movement, and eddy currents in all 660 vol (3 shells * 2 phase-encoding directions * 110 images each). The corrected b0 volume from TOPUP was entered into BET to obtain a brain mask. We used DTIFIT to estimate fractional anisotropy (FA) maps and BEDPOSTX to estimate up to three fibers per voxel using custom settings for multishell data (Behrens et al., 2007, Jbabdi et al., 2012).

#### 2.5.7 Probabilistic tractography

We used PROBTRACKX2 implemented in FSL to estimate connectivity profiles for each 0.8 mm isotropic voxel in the striatum (Behrens et al., 2003b, Behrens et al., 2007). Each voxel was seeded with 10 k streamlines and standard parameter settings. We then extracted connectivity profiles for voxels at coordinates specified by the anatomical masks. This connectivity matrix contained one row for each voxel in the region of interest (e.g. the left caudate nucleus), and one column for each cortical region. As such, this connectivity matrix for a single participant for a single striatal region of interest has dimensions of nVoxels * nTargetRegions. We did not perform more targeted connectivity analysis based on known corticostriatal pathways. The locations of the seed voxels used in probabilistic tractography were recorded and used later to extract functional signals from identical locations.

### 2.6 Regressing functional signals on connectivity profiles

In order to predict function from structure we used a linear regression model. For each of the four separate regions (bilateral caudate nucleus and putamen) we extracted functional signals for foot > hand, reward and expected value contrasts at voxel locations identical to the seed coordinates of the diffusion data. We then used LOOCV to predict functional activation in voxels in participant n based upon the measured relationship between structure and function in the remaining n-1 participants (Fig. 3). All functional data were smoothed at 6 mm FWHM and z-scored within-region before entering the regression, though leaving the data unsmoothed did not drastically alter results in a similar study (Saygin et al., 2012). Similarly, although we collected the dataset at high-resolution, the connectivity profiles are inherently smooth, and some smoothing was appropriate to account for misalignments of the diffusion-weighted and functional images.

The design matrix for each participant contained 149 columns (1 intercept and 148 target regions) and the number of rows corresponded to the number of voxels in the seed region. Each value in the design matrix indicated the number of samples that reached the target region, z-scored across striatal voxels for each target region separately. The dependent variable was each voxel's functional response (expressed as beta coefficient) to a contrast, also z-scored within-subject and within-subregion. The regression coefficients for n-1 participants were averaged and used to predict each voxel's functional response in participant n based on its connectivity profile. This is subtly different from previous applications of this technique whereby data from all voxels from n-1 participants were concatenated into a single predictor matrix (Saygin et al., 2012). Our method for calculating the structure-function relationship weights every participant equally irrespective of number of voxels per participant, whereas the Saygin et al. method linearly weights participants by their number of voxels. Given similar ROI sizes across participants, however, this does not meaningfully affect the results.

We assessed the accuracy of the connectivity model by calculating a Pearson correlation with the observed functional signal. Note we used correlation rather than mean absolute error, which is used in the original paper (Saygin et al., 2012). Given z-scored predictions and observations, these two measures will be highly correlated.

Rather than use the connectivity data, we can also predict a voxel's functional coefficients from the group's functional coefficients (Saygin et al., 2012). The accuracy of these predictions was also assessed using a LOOCV approach, whereby a functional group-average was calculated for n-1 participants in MNI space, transformed to the nth participant's native space and z-scored within-region to predict that participant's functional signals. The accuracy of this prediction was again assessed through Pearson's correlation. We did not directly compare Pearson's r between the two predictive models as this comparison is confounded by data quality. For example, had we ran this experiment with a better signal-to-noise ratio in the diffusion sequence, it would be reasonable to assume our Pearson's r would be higher for the connectivity prediction. We also compared our results to 4-fold (rather than n-fold) cross-validation, and observed no meaningful difference.

We also assessed the *unique* variance captured by the structural and functional group-average predictions, respectively. To do so we orthogonalized one prediction with respect to the other, leaving only the unique variance. For example, to obtain the accuracy of structural connectivity prediction not already accounted for in the functional group-average, we regressed the predicted voxel values from the structural connectivity prediction onto the functional group-average. This was performed using MatLab's glmfit. The residuals are then the orthogonalized prediction, from which all shared variance with the functional group-average has been removed. We used this vector of residuals to correlate with the observed activity values and calculate the Pearson correlation.

### 2.7 Data availability

The datasets generated during and/or analyzed during the current study are available from the corresponding author on reasonable request. The group functional contrast maps are available on NeuroVault (http://neurovault.org/collections/1381/) as are the automated and manual segmentations of basal ganglia structures (http://neurovault.org/collections/1380/). We also provide 9 maps detailing the average observed and predicted signal for the three contrasts under investigation, as well as the mean absolute error (MAE) of the prediction (http://neurovault.org/collections/1381/).

## 3 Results

Participants on average missed 1.4% of trials (SD: 1.0). The reaction time for hand responses was 683 ms (SD: 37 ms) and 723 (SD: 46) for foot responses (foot - hand, mean across subjects = 40 ms, 95% CI of difference ± 27 ms, Cohen's d = 0.63). Participants received a reward on average on 51% (SD: 0.054) of trials, where random play would yield 48% reward – most likely reflecting the high level of difficulty due to volatile reward probabilities.

We fit a reinforcement learning model to participants’ choices based on their past actions and rewards. We used this model to calculate expected outcome values for each trial, and entered these into a model-based fMRI analysis along with action and reward to index how strongly the BOLD signal in each voxel co-varied with these variables. The Bayesian model comparison and winning model parameters are described in Table 3, Table 4, respectively.

**Table 3:** Model comparison results with only the five best models shown here. Each reinforcement learning model had a single learning rate and inverse temperature parameter. Added to this base model was perseverance, effector bias, separate learning rate for positive and negative feedback (‘neg α’), a lapse rate, and exponential decay for unchosen options back to Q=0.5. The integrated Bayesian Information Criterion was estimated for 200 k samples each from the practice and scanning session, and summed over both sessions and participants to arrive at final BICi.

Additional parameters	BICi	δBICi
neg α, decay	12393	0
perseverance, neg α, decay	12400	+7
lapse rate, neg α, decay	12427	+34
perseverance, lapse rate, neg α, decay	12435	+42

**Table 4:** Parameter estimates from winning model for the scanning session.

Parameter	25th percentile	median	75th percentile
Positive learning rate	0.54	0.61	0.72
Negative learning rate	0.20	0.32	0.38
Inverse temperature	3.12	5.01	5.87
Decay	0.36	0.55	0.73

**Fig. 2:**
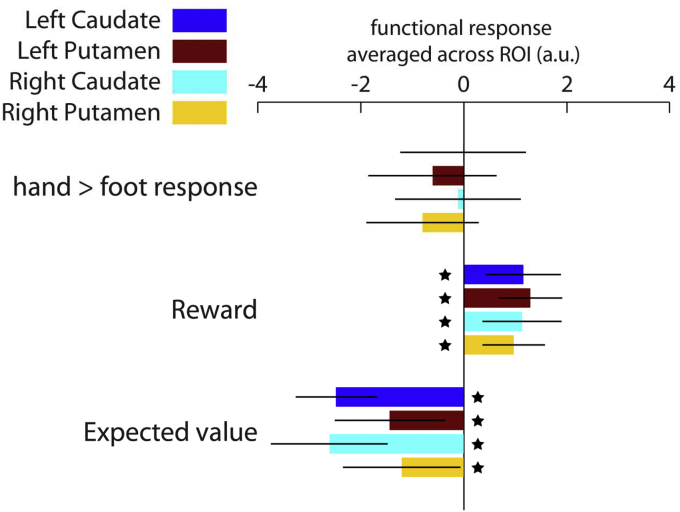
Extracted functional coefficients from anatomically defined bilateral putamen and caudate nucleus. None of the 4 regions showed modulation by hand versus foot motoric responses. As observed previously, these regions showed a positive response to rewards and a negative response to expected value at the time of outcome. These signals show features consistent with a reward prediction error (RPE). Error bars indicate 95% CI. Stars indicate p < 0.05 for 1-sample *t*-test against zero, uncorrected for multiple comparisons.

As expected, the average contrast values from anatomical ROIs in each participant's native space reflected both reward and expectation signals (Fig. 2). Activity in the striatum is known to reflect reward prediction error (RPE), calculated as reward minus expectation. A region encoding a RPE should, in terms of average BOLD response across voxels, show a positive effect of reward and a negative effect of expectation. We observed this pattern in both the putamen (reward: left, p = 0.0002, right, p = 0.003; expected value at outcome: left, p = 0.01, right, p = 0.04; uncorrected for multiple comparisons) and caudate nucleus (reward: left, p = 0.004, right, p = 0.006; expected value at outcome: left, p = 1 × 10^−6^, right, p = 9 × 10^−5^; uncorrected for multiple comparisons). We also compared the response to hand and foot actions without reference to value. No striatal region showed a significantly greater response to one or other of these effectors. As a positive control we examined the cerebellum as the motor cortex was outside our restricted fMRI volume. We observed higher BOLD activity on statistical parametric maps for foot compared to hand actions in the anterior right cerebellum, and higher activity for hand compared to foot actions just posterior to this location, consistent with the known anatomy of the cerebellum (Buckner, 2013).

**Fig. 3:**
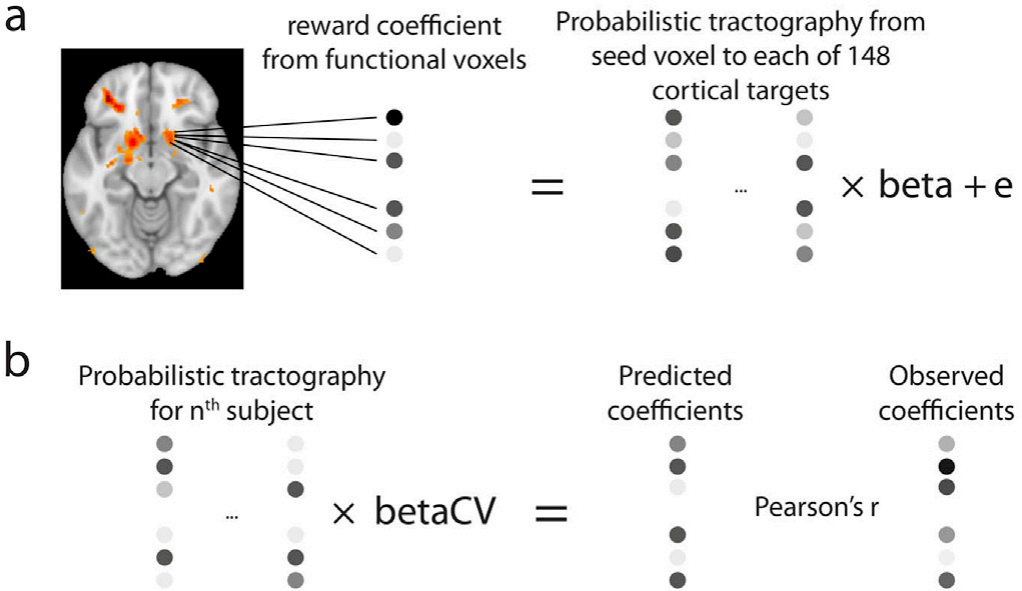
Overview of the leave-one-out cross-validation regression approach. (a) For each participant we estimated regression coefficients (‘beta’) describing how functional activity related to structural connectivity with 148 cortical targets. This was implemented for each striatal subregion and functional contrast independently. (b) The regression coefficients were averaged across the n-1 participants (‘betaCV’ indicating cross-validated beta) and multiplied by the n-th subject's connectivity matrix to predict the contrast coefficient in each voxel. The Pearson correlation between predicted and observed coefficients was recorded and the approach repeated for each participant, yielding n correlation coefficients for each contrast and striatal subregion. A Pearson's r significantly greater than zero indicates differences in functional responses between voxels are predicted from differences in structural connectivity.

The existence of focal corticostriatal projections suggests local variation in functional activity within each of 4 ROIs from Fig. 2. If true, this local variation might be partly explained by local differences in corticostriatal connectivity. We used a regression-based method first introduced by (Saygin et al., 2012) to test such a relationship between structural connectivity and function (Fig. 3). In addition to a prediction based on structural connectivity, we also used the functional group-average as a benchmark predictive model by calculating the average group response in each voxel based on n-1 subjects, and using this as a prediction for subject n. Consequently, this approach tests consistency, across subjects, in the spatial distribution of functional responses irrespective of structural connectivity.

**Fig. 4:**
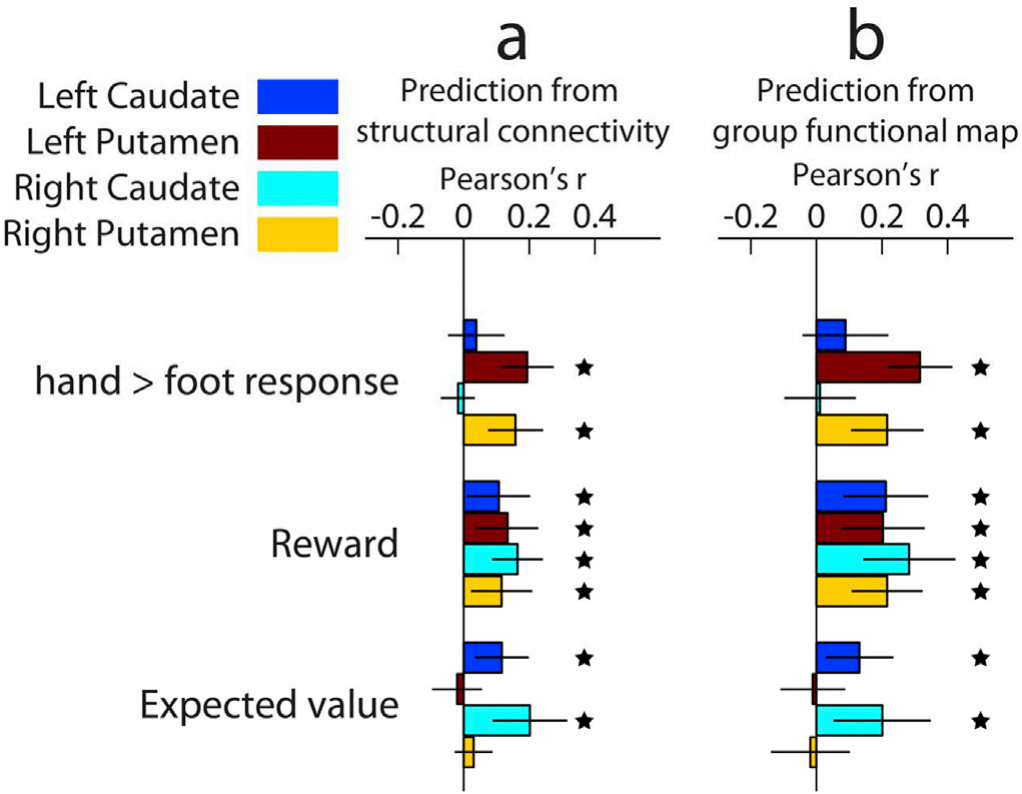
Accuracy of predictions from the connectivity model and the functional group-average model. (a) The structural connectivity model predicts the functional contrast value for each voxel in an ROI from corticostriatal connectivity of that voxel. Despite none of the 4 ROIs showing an average effect of the hand > foot response contrast, local activity in bilateral putamen but not caudate nucleus can be predicted from structural connectivity. Local variation in reward contrast values could be predicted in each of the 4 ROIs. In contrast, local variation in expected value responses could only be predicted in bilateral caudate nucleus but not putamen – despite each ROIs showing an average effect of expected value. (b) Instead of using corticostriatal connectivity to predict function, we also used the functional group-average to predict activity. The performance of this model shows a similar pattern to the structural connectivity model. Error bars indicate 95% CI. Stars indicate p < 0.05 for 1-sample *t*-test against zero, uncorrected for multiple comparisons.

We observed a pattern of results that was similar between the connectivity and group predictive models, and identical for left and right hemispheres (Fig. 4; statistics in Table 5). Whereas none of the regions on average showed significant differential activity for hand versus foot actions (Fig. 2), local variation in this signal could be predicted from connectivity patterns in the putamen but not caudate nucleus (Fig. 4a). Additionally, local reward signals could be predicted from cortical connectivity in bilateral putamen as well as in caudate nucleus. In contrast, local variation in activity related to expected value—which was significantly represented in the average response of each of the ROIs (Fig. 2)—was only predicted from corticostriatal connectivity in bilateral caudate nucleus but not in the putamen. A group average of the observed and predicted maps for each contrast, as well as the MAE of the prediction from corticostriatal connectivity, are available online (http://neurovault.org/collections/1381/). Overall, these patterns of results demonstrate that a relationship between corticostriatal connectivity and striatal function is reliable across subjects.

**Fig. 5:**
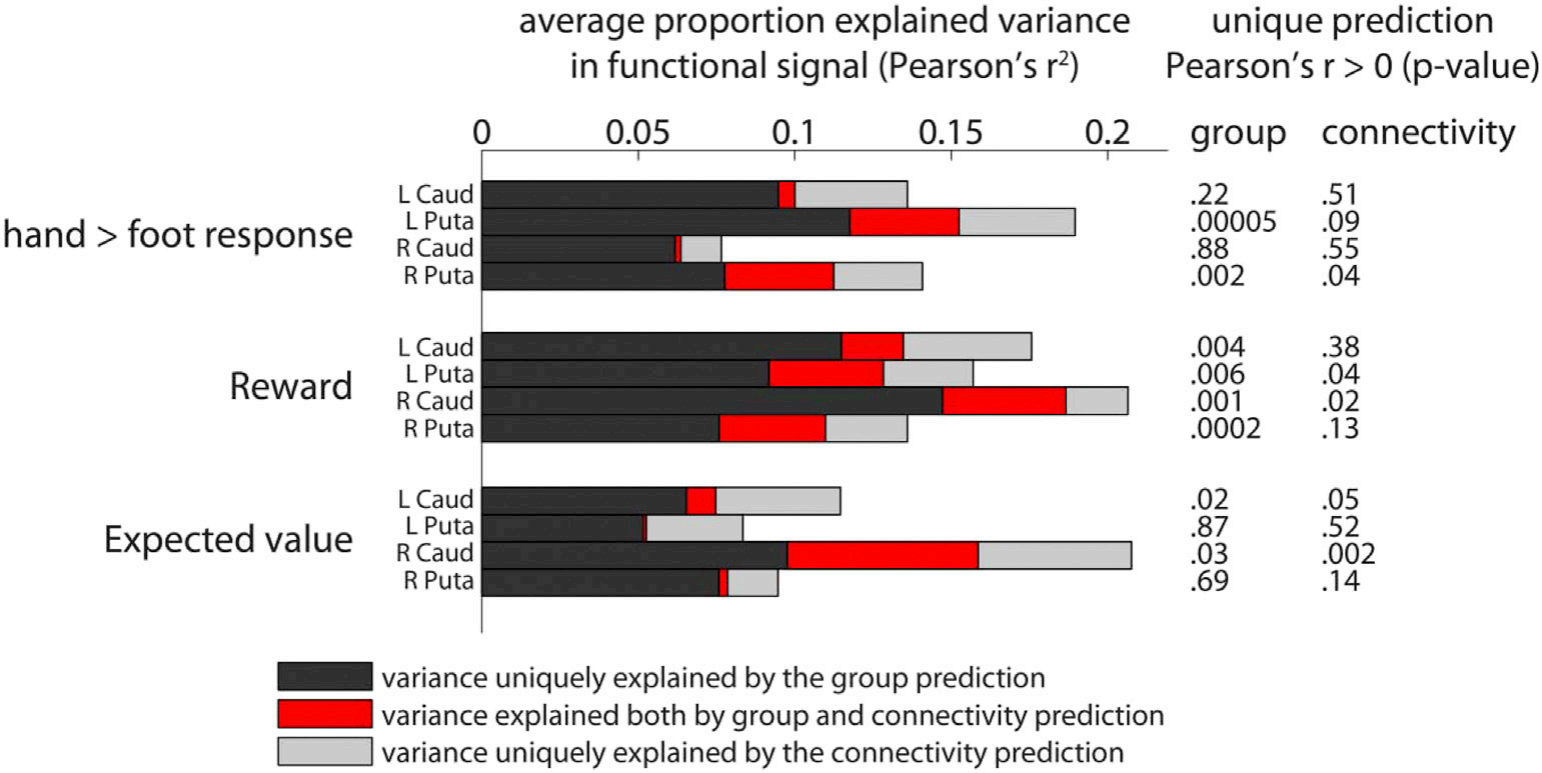
Quantifying explained variance in the functional signal by predictive models. The similarity in prediction performance for connectivity and functional group-average predictions (Fig. 4) raised the question whether the connectivity model is simply capturing spatial patterns of activity already explained by the functional group-average. To formally test this, we orthogonalized the connectivity and group prediction with respect to one another and regressed the observed signal on these orthogonalized predictions. This revealed variance in the functional signal uniquely explained by the group prediction (black) and the connectivity prediction (light grey). We also estimated how much overlap in explained variance there was between both models, shown in red. Note that despite some overlap (red), the majority of explained variance is uniquely attributed to the group or connectivity predictions, indicating the prediction from connectivity did not merely recapitulate the functional group-average. The p-values represent a 1-sample *t*-test against zero for the Pearson's correlations between orthogonalized prediction and observed value, uncorrected for multiple comparisons.

**Table 5:** Statistics for Fig. 4, Fig. 5. Values represent Pearson's r expressed as the mean and 95% CI across participants. P-value is from a *t*-test of r against zero, uncorrected for multiple comparisons. The column ‘full’ represents the correlation when the prediction is not competing for variance with the alternative prediction. ‘Orthogonalized’ refers to the performance of the prediction after orthogonalizing the prediction with respect to the alternative prediction method.

Contrast	Region	Connectivity prediction		Functional group-average prediction	
		Full	Orthogonalized	Full	Orthogonalized
Hand > foot	L Caudate	0.04 [-0.05, 0.12] p = 0.37	0.026 [-0.05, 0.11] p = 0.51	0.04 [-0.04, 0.22] p = 0.18	0.078 [-0.05, 0.21] p = 0.22
	L Putamen	0.19 [0.11, 0.27] p = 0.00005	0.066 [-0.01, 0.14] p = 0.09	0.19 [0.22, 0.41] p < 0.00001	0.247 [0.15, 0.35] p = 0.00005
	R Caudate	−0.02 [-0.07, 0.03] p = 0.48	−0.014 [-0.06, 0.03] p = 0.55	−0.02 [-0.10, 0.12] p = 0.83	0.008 [-0.10, 0.12] p = 0.88
	R Putamen	0.16 [0.07, 0.24] p = 0.0008	0.068 [0.00, 0.13] p = 0.04	0.16 [0.11, 0.33] p = 0.0005	0.162 [0.06, 0.26] p = 0.002
Reward	L Caudate	0.11 [0.01, 0.20] p = 0.03	0.037 [-0.05, 0.12] p = 0.38	0.11 [0.08, 0.34] p = 0.003	0.190 [0.07, 0.31] p = 0.004
	L Putamen	0.13 [0.04, 0.23] p = 0.008	0.069 [0.00, 0.14] p = 0.04	0.13 [0.08, 0.33] p = 0.003	0.161 [0.05, 0.27] p = 0.006
	R Caudate	0.16 [0.09, 0.24] p = 0.0002	0.066 [0.01, 0.12] p = 0.02	0.16 [0.14, 0.42] p = 0.0004	0.232 [0.10, 0.36] p = 0.001
	R Putamen	0.12 [0.02, 0.21] p = 0.02	0.051 [-0.02, 0.12] p = 0.13	0.12 [0.11, 0.32] p = 0.0004	0.186 [0.10, 0.27] p = 0.0002
Expected value	L Caudate	0.12 [0.03, 0.20] p = 0.007	0.081 [0.00, 0.16] p = 0.04	0.12 [0.03, 0.23] p = 0.02	0.116 [0.02, 0.21] p = 0.02
	L Putamen	−0.02 [-0.10, 0.06] p = 0.58	−0.023 [-0.10, 0.05] p = 0.52	−0.02 [-0.11, 0.09] p = 0.82	−0.008 [-0.11, 0.09] p = 0.87
	R Caudate	0.20 [0.09, 0.32] p = 0.001	0.128 [0.05, 0.21] p = 0.002	0.20 [0.05, 0.35] p = 0.01	0.135 [0.01, 0.26] p = 0.03
	R Putamen	0.03 [-0.03, 0.09] p = 0.29	0.038 [-0.01, 0.09] p = 0.14	0.03 [-0.14, 0.10] p = 0.75	−0.023 [-0.14, 0.10] p = 0.69

However, we could similarly predict functional responses in voxels based on the functional group-average (Fig. 4b). This invites the question as to the extent the connectivity profile captures a unique component of function, or whether it explains the same variance in functional signals already captured by the functional group-average. To test this, we calculated the proportion of variance explained in the functional response by 1) the connectivity prediction orthogonalized with respect to the group prediction (i.e. capturing unique variance explained by connectivity); 2) the group prediction orthogonalized with respect to the connectivity prediction (containing unique variance captured by the functional group-average model only); 3) the connectivity and group prediction as two predictors in a single regression (each prediction's unique variance + shared variance). The variance in the functional response shared between the group and connectivity prediction is then the total variance (#3) minus the sum of unique variances from the connectivity and group models (#1 + #2). The contributions to explained variance of the two models as well as their shared variance are shown in Fig. 5.

To test whether the orthogonalized predictions performed better than chance, we report the same analysis as in Fig. 4—where we test the Pearson correlation against zero—but now using orthogonalized predictions (Fig. 5; Table 5). The signal uniquely explained by the connectivity prediction remained significantly greater than zero in each of the regions observed originally, with the sole exception of the hand > foot response in left putamen. Thus, even when removing any variance explained by consistent functional activations across the group, corticostriatal connectivity drives unique functional responses voxel-by-voxel within striatal regions.

## 4 Discussion

In this study we used a reinforcement learning task to elicit BOLD responses in the striatum related to actions, rewards and expected value. The aim was to examine whether local variation in these responses could be predicted from local variation in corticostriatal connectivity. We found that the *average* activity in both caudate nucleus and putamen varied with reward and expected value, but did not differentiate between effectors used to perform an action. Corticostriatal connectivity predicted voxel-specific responses to reward in both putamen and caudate nucleus, predicted expected value in caudate nucleus but not putamen, and predicted effector-specific activity in the putamen but not caudate nucleus. These results support the widely held belief that partially distinct functional zones in the striatum are, at least in part, determined by the patterns of anatomical inputs they receive from cortex (Alexander et al., 1986, Averbeck et al., 2014, Draganski et al., 2008, Haber, 2003, Haber and Behrens, 2014).

A goal of this study was to understand how striatal functional signals arise from cortical inputs. The notion that anatomical connectivity determines function is common in neuroscience. In the striatum cell populations with projections along the direct and indirect pathway have distinct functional roles in movement (Cui et al., 2013, Kravitz et al., 2010, Yttri and Dudman, 2016) and in reinforcement learning (Kravitz et al., 2012). In humans, connectivity fingerprints have been used to segment individual brain structures with remarkable similarity to functional zones (Behrens et al., 2003a, Johansen-Berg et al., 2004). This same technique has revealed anatomical parcellation of the striatum (Draganski et al., 2008, Georgiou-Karistianis et al., 2011, Tziortzi et al., 2014, Verstynen et al., 2012), but this parcellation has not been directly linked to patterns of functional activations.

We used a recently introduced analytical method (Osher et al., 2015, Saygin et al., 2012), comprising a cross-validation technique to assess the predictive power that connectivity has over functional signals. We adjusted their approach to avoid two issues of concern. Firstly, we used the Pearson correlation rather than mean absolute error (MAE) to assess predictive accuracy. Whereas the correlation is insensitive to the variance of the signals, the MAE is linearly related to the standard deviation of the signals. This can lead to spurious differences between group and connectivity depending on the point in the analysis pipeline that the group and connectivity predictions are normalized—though this is not the case in the original work (Saygin et al., 2012). Secondly, we avoided the use of statistical tests at the voxel level to compare models to one another or to chance. As voxels are not independent measurements due to inherent smoothness in the signal, tests such as permutation and t-tests as performed in the original paper are invalid (Breakspear et al., 2004, Nichols and Holmes, 2002). We therefore only performed tests on summary statistics of each participant.

We observed that the *average* functional response in a striatal region does not necessarily predict whether or not differential activity *within* that region is predicted from functional group-averages or structural connectivity. For example, although the left and right putamen on average showed no differential BOLD response for hand versus foot actions, *within* the putamen there was a pattern of activity, consistent across participants, that differentiated between such actions. This is unsurprising given the existence of somatotopic motor loops (Nambu, 2011, Nambu et al., 2002) and, more generally, topographically organized corticostriatal loops (Haber and Knutson, 2009). More surprisingly, the putamen had a lower BOLD response with increasing expected value *on average*, but this signal lacked spatial consistency and bore no observable relationship to connectivity. This raises a possibility that signals in the striatum might be divided into two components: one driven by local connectivity, and one which is diffuse and non-specific. This is similar to the notion of global inhibition and selective dis-inhibition in striatal pathways (Frank, 2011, Mink, 1996). If correct, such a functional architecture would have important implications for the way in which computations are performed by the striatum.

The results presented here should be understood in light of a number of limitations. Firstly, the statistics are uncorrected for multiple comparisons, and not all findings would survive Bonferroni correction. The main argument against these findings being false positives is the fact that the majority of results replicate between left and right hemisphere. This is highly unlikely under the null hypothesis, and therefore strengthens our confidence in the reported findings. Secondly, we have not examined whether an individual's structure-function relationship relates to reinforcement learning parameters. Others have observed that corticostriatal projections can predict reinforcement learning parameters (de Wit et al., 2012, Verstynen, 2014). Similarly, we might hypothesize that the individual differences in how strongly specific cortical regions drive striatal activity would also be reflected in reinforcement learning parameters. This question would be best studied in larger datasets than that presented here, for example in the Human Connectome Project dataset (Van Essen et al., 2012).

Our results have implications for the standard practice of averaging physiological signals across the caudate nucleus or putamen, as doing so will discard a considerable amount of local heterogeneity in function. One way to parcelate the striatum is through diffusion-weighted imaging alone, as done before (Draganski et al., 2008, Lehericy et al., 2005, Verstynen et al., 2012). Other work has examined how overlap between corticostriatal projections relates to functional signals (Verstynen, 2014). The work presented here took a data-driven approach to predicting functional signals in the striatum from a full corticostriatal connectivity profile, i.e. we did not single out specific corticostriatal pathways to assess their relationship to function. This contrasts with recent work that focused on frontostriatal and parietostriatal connectivity, observing ‘convergence zones’ in the striatum that were confirmed by resting state functionality connectivity (Jarbo and Verstynen, 2015). Indeed, resting state functional connectivity has been a popular tool to understand corticostriatal connectivity, given the inherent limitations in diffusion imaging reveal the intermeshed pathways that reach deep into the basal ganglia (Choi et al., 2012, Jung et al., 2014, Kim et al., 2013, Tavor et al., 2016). Others still have used meta-analyses of functional imaging studies to reveal five distinct striatal zones that show BOLD changes for specific psychological processes, as well as reveal corticostriatal networks involved in cognition (Pauli et al., 2016).

The further development of these approaches is likely to improve our ability to map an individual's corticostriatal system using non-invasive methods.

## Conflicts of interest

The authors declare no competing financial interests.
